# Supplementary material for: The Association Between Alpha-1 Adrenergic Receptor Antagonists and In-Hospital Mortality from COVID-19
Source: medRxiv. 2021 Feb 11:2020.12.18.20248346. Originally published 2020 Dec 22. Preprint. [Version 2] doi: 10.1101/2020.12.18.20248346 (PMC7781337; doi:10.1101/2020.12.18.20248346)
Supplement: 1 [file NIHPP2020.12.18.20248346-supplement-1.pdf]

medRxiv preprint doi: https://doi.org/10.1101/2020.12.18.20248346; this version posted February 11, 2021. The copyright holder for this preprint
(which was not certified by peer review) is the author/funder, who has granted medRxiv a license to display the preprint in perpetuity.
It is made available under a CC-BY 4.0 International license .

Supplementary Figure 1. Vital Signs at Time of Admission

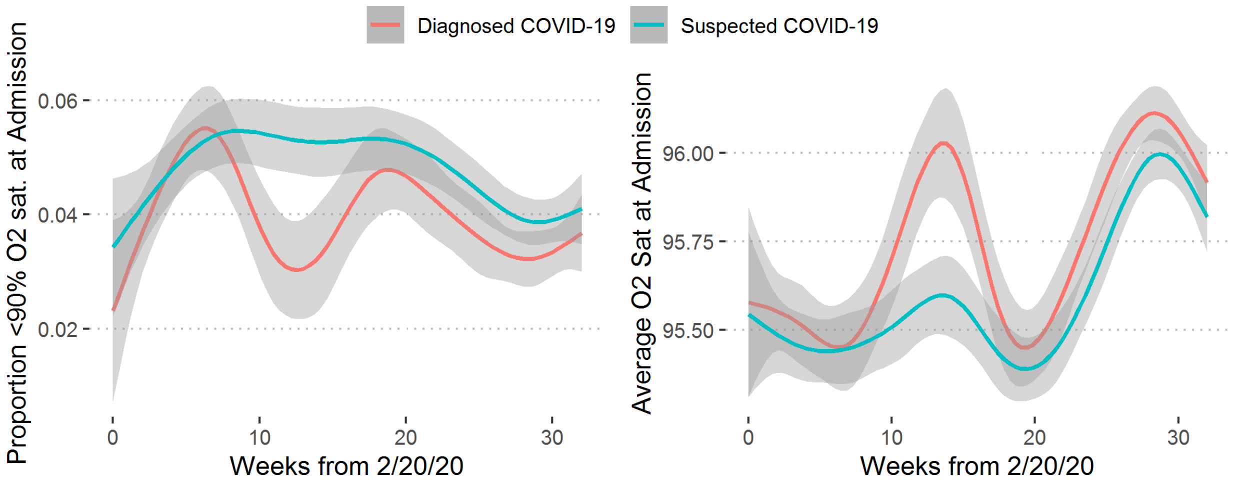

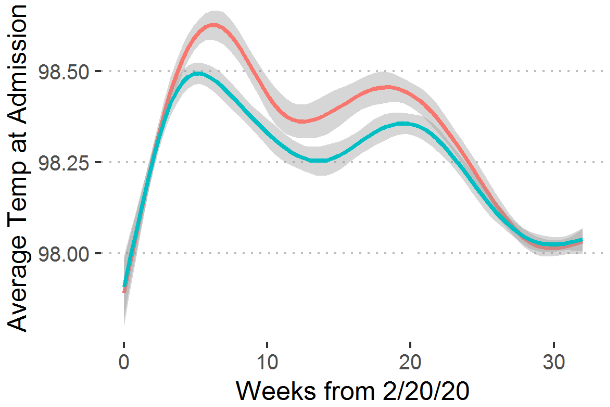

Measure	Diagnosed COVID-19	Suspected COVID-19	Difference
O2 Sat <90%	0.038	0.046	-0.007
Average O2 Sat	95.816	95.672	0.144
Average Temperature	98.233	98.211	0.023

The diagrams show vital signs for patients diagnosed with COVID-19 (red line) and an expanded cohort of patients with suspected COVID-19 (blue line). Smoothed lines are from a LOESS model with 95% confidence intervals shown (gray ribbons).

medRxiv preprint doi: https://doi.org/10.1101/2020.12.18.20248346; this version posted February 11, 2021. The copyright holder for this preprint (which was not certified by peer review) is the author/funder, who has granted medRxiv a license to display the preprint in perpetuity.
It is made available under a CC-BY 4.0 International license .

Supplementary Figure 2. In-hospital and 28-Day Mortality by Use of Tamsulosin at Time of Hospital Admission with COVID-19.

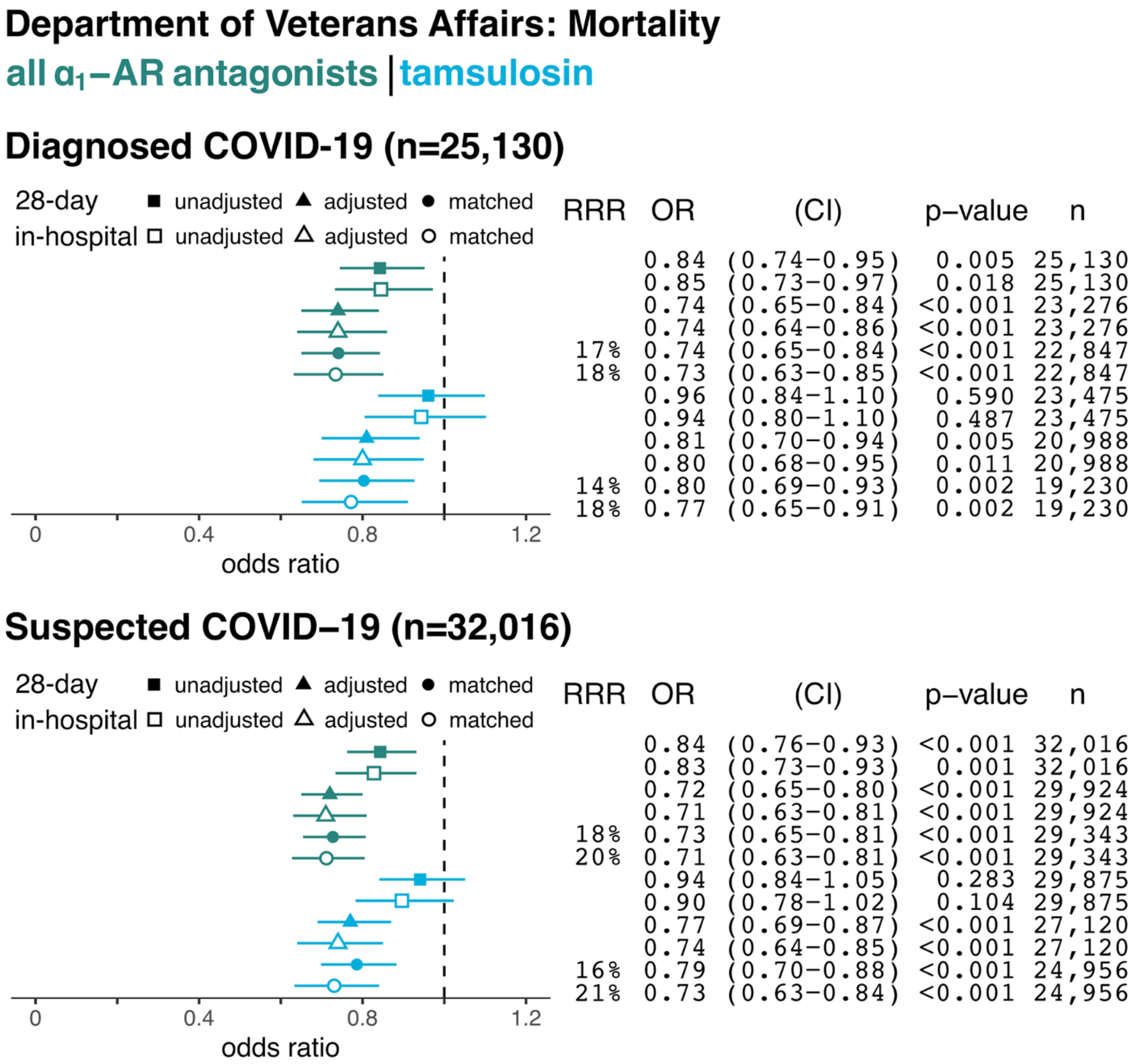

Data are shown for hospitalized patients diagnosed with confirmed COVID-19 (top panel) and with confirmed plus suspected COVID-19 (bottom panel). Forest plots showing odds ratios (OR) of in-hospital mortality based on prior use of any alpha-1 adrenergic receptor antagonists (dark green) or tamsulosin (light blue) in each panel. Relative risk reduction (RRR), odds ratios (ORs) for death, 95% confidence intervals (CI), and p-values (for unadjusted, adjusted, and matched models), and sample size (n) for each analysis are shown on the right.

medRxiv preprint doi: https://doi.org/10.1101/2020.12.18.20248346; this version posted February 11, 2021. The copyright holder for this preprint (which was not certified by peer review) is the author/funder, who has granted medRxiv a license to display the preprint in perpetuity. It is made available under a CC-BY 4.0 International license .

Supplementary Figure 3. Adjusted Odds of In-hospital Mortality and Use of α1-AR Antagonists by Week

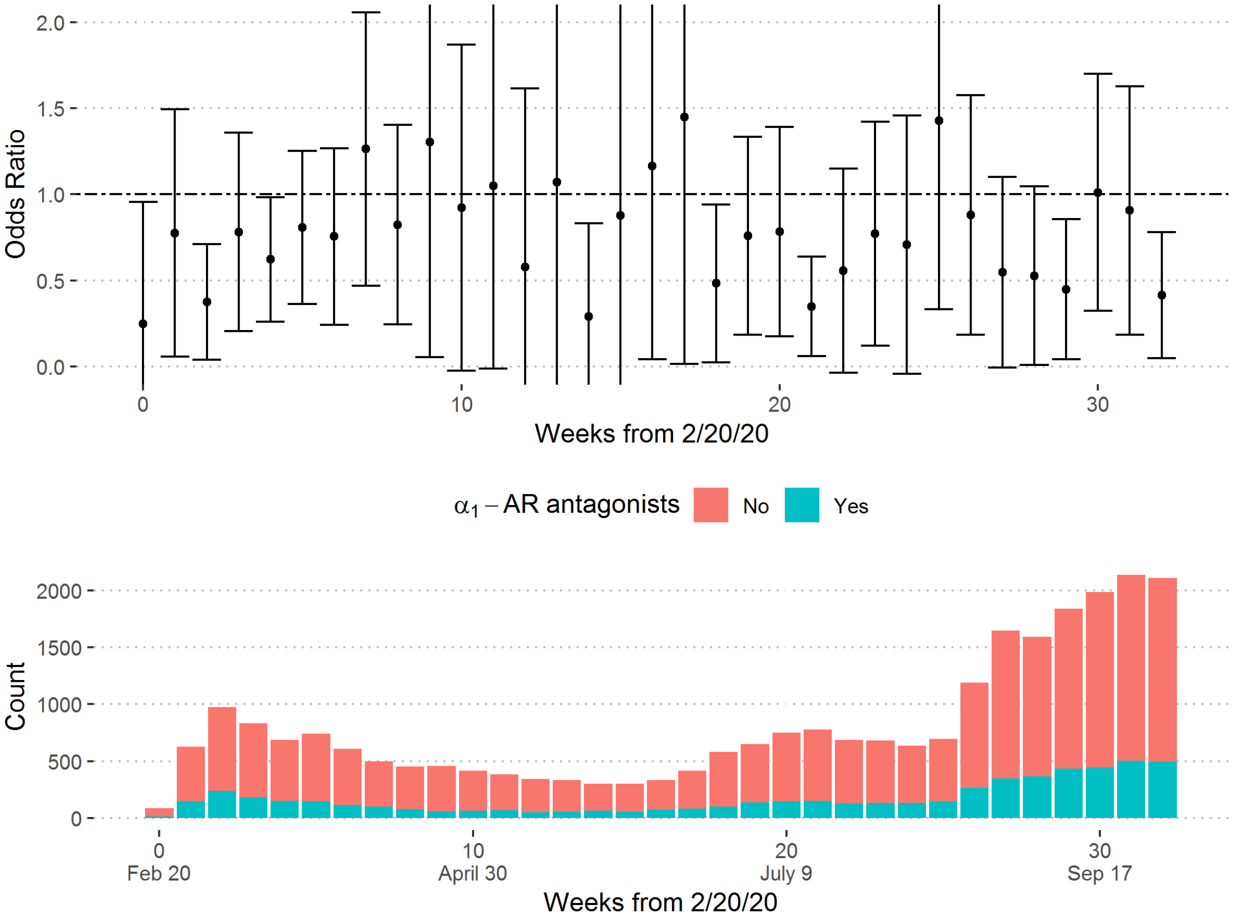

Top panel shows adjusted odds ratios of in-hospital mortality and use of α1-AR antagonists by week of admission. Top panel truncated between 0 and 2 to aid visualization. Bottom panel shows number of new admissions by week and use of α1-AR antagonists (bottom).

medRxiv preprint doi: https://doi.org/10.1101/2020.12.18.20248346; this version posted February 11, 2021. The copyright holder for this preprint (which was not certified by peer review) is the author/funder, who has granted medRxiv a license to display the preprint in perpetuity.
It is made available under a CC-BY 4.0 International license .

Supplementary Figure 4. Adjusted Odds of In-hospital Mortality and Use of α_1-AR Antagonists by VA Station

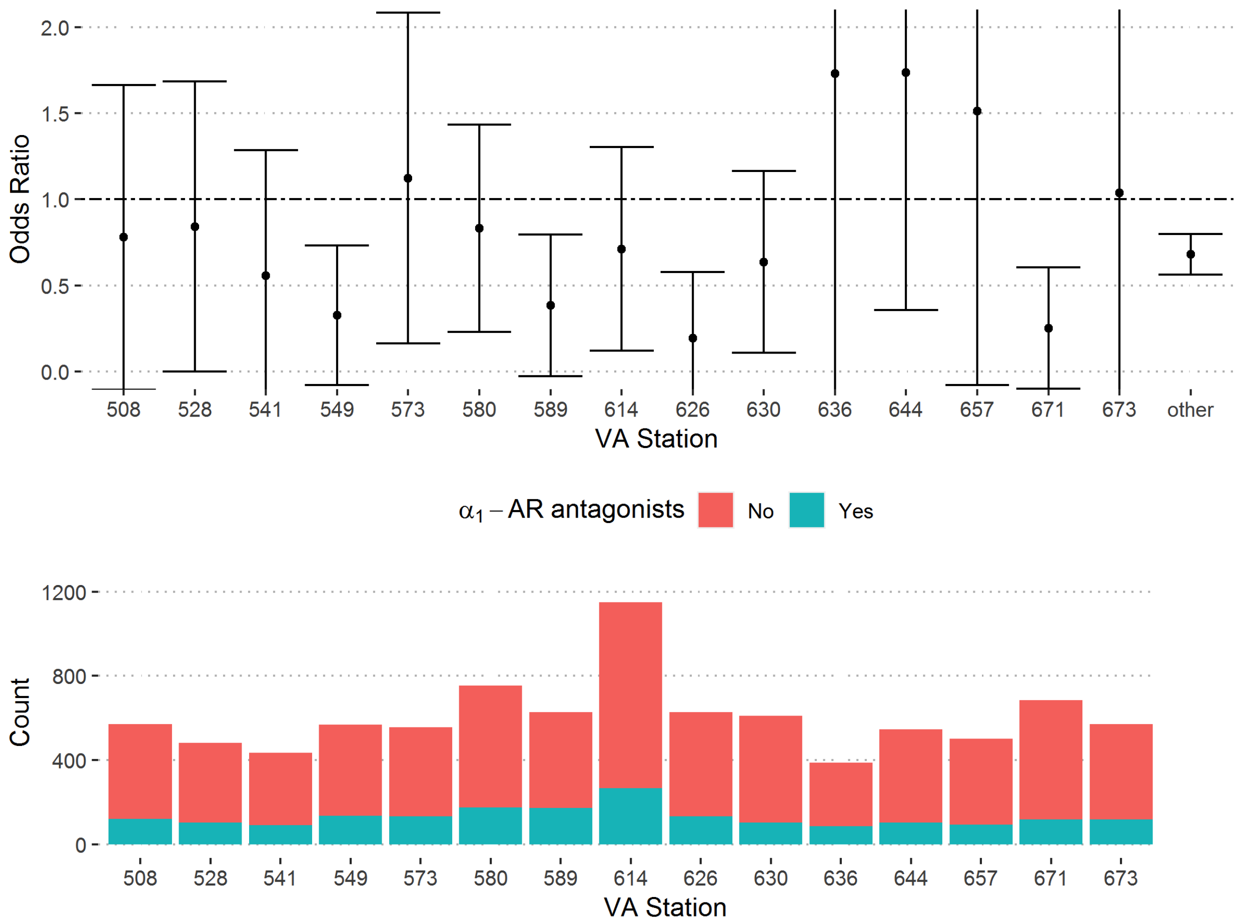

Top panel shows adjusted odds ratios of in-hospital mortality in patients taking α_1-AR antagonists by VA station. Top panel truncated between 0 and 2 to aid visualization. Bottom panel shows number of new admissions and use of α_1-AR antagonists by VA station (bottom). For other VA stations, the number of admissions of patients not using α_1-AR antagonists was 7,645 and number of admissions of patients using α_1-AR antagonists was 1,845. VA stations shown: 508 = Atlanta, 549 = Dallas, 573 = Gainesville, 580 = Houston, 589 = Kansas City, 614 = Memphis, 630 = New York Harbor, 644 = Phoenix, 671 = San Antonio, 673 = Tampa.
